# Combination of NK Cells and Cetuximab to Enhance Anti-Tumor Responses in RAS Mutant Metastatic Colorectal Cancer

**DOI:** 10.1371/journal.pone.0157830

**Published:** 2016-06-17

**Authors:** John Pradeep Veluchamy, Jan Spanholtz, Marleen Tordoir, Victor L. Thijssen, Daniëlle A. M. Heideman, Henk M. W. Verheul, Tanja D. de Gruijl, Hans J. van der Vliet

**Affiliations:** 1 Department of Medical Oncology, VU University Medical Center, Cancer Center Amsterdam, Amsterdam, The Netherlands; 2 Department of Pathology, VU University Medical Center, Amsterdam, The Netherlands; 3 Glycostem Therapeutics, Oss, The Netherlands; Istituto Superiore di Sanità, ITALY

## Abstract

The ability of Natural Killer (NK) cells to kill tumor targets has been extensively studied in various hematological malignancies. However, NK cell therapy directed against solid tumors is still in early development. Epidermal Growth Factor Receptor (EGFR) targeted therapies using monoclonal antibodies (mAbs) such as cetuximab and panitumumab are widely used for the treatment of metastatic colorectal cancer (mCRC). Still, the clinical efficacy of this treatment is hampered by mutations in RAS gene, allowing tumors to escape from anti-EGFR mAb therapy. It is well established that NK cells kill tumor cells by natural cytotoxicity and can in addition be activated upon binding of IgG_1_ mAbs through Fc receptors (CD16/FcγRIIIa) on their surface, thereby mediating antibody dependent cellular cytotoxicity (ADCC). In the current study, activated Peripheral Blood NK cells (PBNK) were combined with anti-EGFR mAbs to study their effect on the killing of EGFR^+/-^ cancer cell lines, including those with RAS mutations. *In vitro* cytotoxicity experiments using colon cancer primary tumors and cell lines COLO320, Caco-2, SW620, SW480 and HT-29, demonstrated that PBNK cells are cytotoxic for a range of tumor cells, regardless of EGFR, RAS or BRAF status and at low E:T ratios. Cetuximab enhanced the cytotoxic activity of NK cells on EGFR^+^ tumor cells (either RAS^wt^, RAS^mut^ or BRAF^mut^) in a CD16 dependent manner, whereas it could not increase the killing of EGFR^-^ COLO320. Our study provides a rationale to strengthen NK cell immunotherapy through a combination with cetuximab for RAS and BRAF mutant mCRC patients.

## Introduction

Epidermal Growth Factor Receptor (EGFR) is expressed on cell surfaces in normal tissues and binding to its ligands activates two important pathways, the RAS-RAF-MAPK and PI3K-PTEN-AKT pathway, which both control cell proliferation, survival and motility [1]. Dysregulation of the EGFR signaling cascade can result in rapid cell division ultimately supporting tumor growth. Several solid tumors show elevated EGFR expression levels, which were shown to be related to poor prognosis [2]. Cetuximab (IgG_1_ chimeric) and panitumumab (IgG_2_ fully humanized) are clinically approved anti-EGFR mAbs that bind to the extracellular domain of EGFR thereby blocking EGFR dimerization, resulting in apoptosis and preventing tumor growth [3].

Regrettably, mutations in the EGFR downstream signaling pathway (e.g. RAS mutations), can lead to constitutive RAS signaling, resulting in unresponsiveness to anti-EGFR therapy [4–6].The fact that in about 40% of patients with metastatic colorectal cancer (mCRC) mutations in the RAS gene can be observed, means that anti-EGFR therapy is applicable in only half of the mCRC patients [7]. Therefore several approaches have been proposed and are currently tested to increase the efficacy of anti-EGFR mAb therapy by overcoming the inhibitory effect of RAS mutation, e.g. by immune effector cell-mediated antibody dependent cell-mediated cytotoxicity (ADCC) [8, 9].

Several immune effector cells in the body have the ability to recognize target molecules on the tumor cell surface, like EGFR on CRC cells, through their FcR-mediated binding of antibodies directed against these targets, leading to potent antitumor immunity. However, due to cytotoxic treatment regimens in solid tumor patients, the immune system can be temporarily dysfunctional, signified by a decrease in immune effector cell subsets [10, 11]. This limitation may be overcome by cellular immunotherapy, such as the adoptive transfer of activated cytolytic Natural Killer (NK) cells. NK cells are part of the innate immune defense, with the ability to kill tumor cells. NK cells comprise of two subsets, from which the majority (about 90%) are phenotypically CD56^dim^ CD16^bright^ and exert mainly cytolytic functions, whereas the other subset of CD56^bright^ CD16^dim^ NK cells primarily exert immune regulatory functions [12]. CD16a (FcγRIIIa), a low affinity Fc receptor, preferably binds to IgG_1_ antibodies and can actively mediate ADCC [13, 14].

This study aims to prove that NK cells are able to induce strong ADCC responses in combination with therapeutic EGFR-targeting mAbs and can thereby overcome the potential limitations of stand-alone anti-EGFR therapy. Therefore, activated PBNK cells were combined with cetuximab or panitumumab to test their ADCC efficacy on EGFR^+^/^-^, RAS^wt^/^mut^, BRAF^mut^ cell lines and primary tumor cells from patients with CRC.

## Results

### More potent NK effector cell activation and ADCC effected by cetuximab than by panitumumab

**Fig 1:**
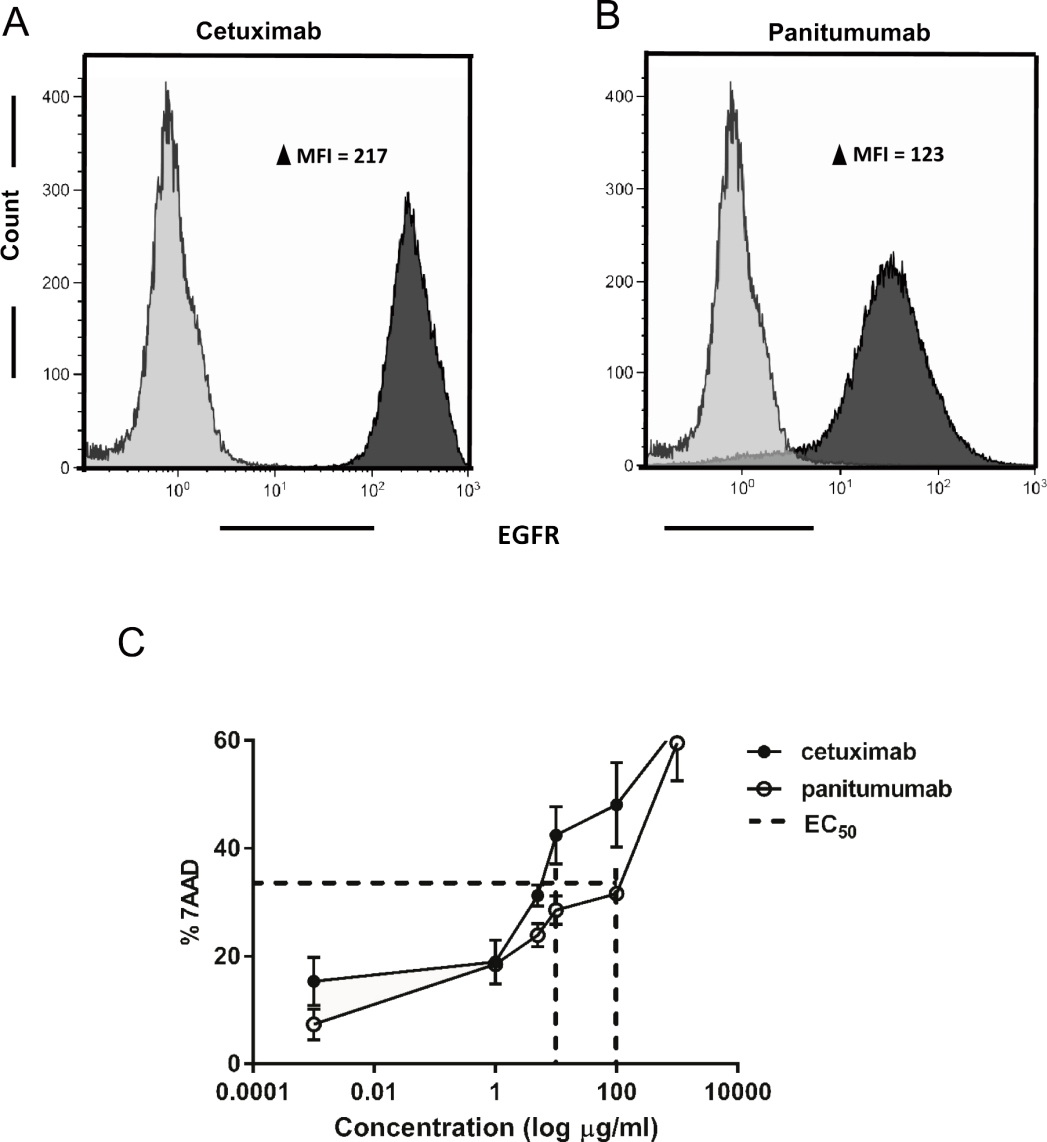
Cetuximab and panitumumab binding affinities and their cytotoxicity towards EGFR^+++^ tumor targets. EGFR overexpressing A431 cells were used to test anti-EGFR mAbs. A and B, Histograms showing binding of 100μg/ml of biotinylated cetuximab (A) and panitumumab (B) to A431 cells. Grey shades represents the streptavidin APC control, black shades represent binding of the biotinylated mAbs. (C) Dose response curve to measure EC_50_ concentration for cetuximab and panitumumab. A431 cells treated with cetuximab and panitumumab at concentrations of 1ng, 1μg, 5μg, 10μg, 100μg and 1000μg per ml for 4 hrs were analyzed for target cell death.

**Fig 2:**
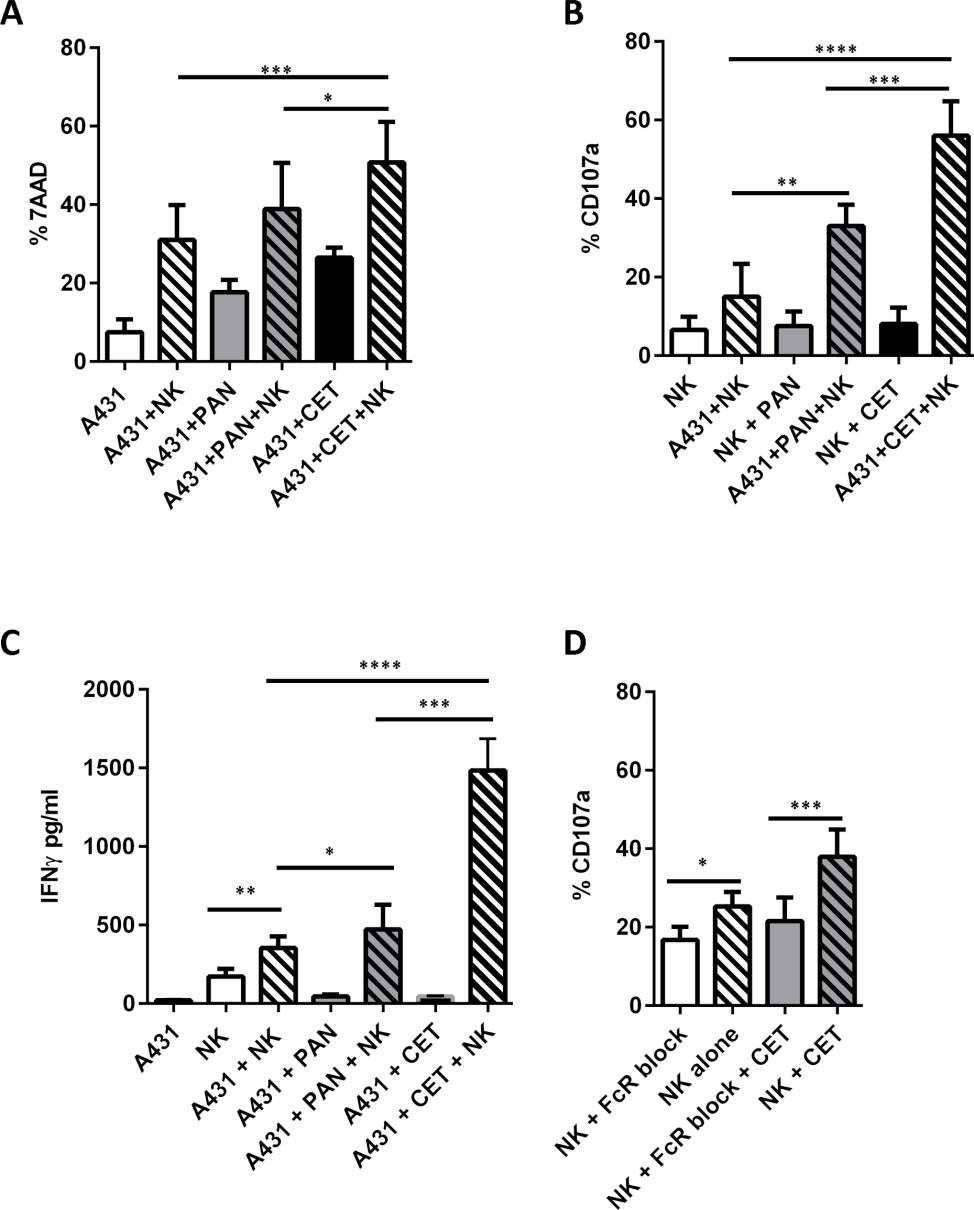
Anti-EGFR mAb cytotoxicity in combination with NK cells. The effect of IL-2 and IL-15 activated PBNK cells, cetuximab and panitumumab on lysis of A431 tumor cells was assessed. The percentage of ADCC was calculated based on percentage of PBSE labelled A431 cells staining positive for 7AAD in different co-culture conditions (A). Effector cells from the assay were stained with CD107a to measure NK cell degranulation by flow cytometry (B). Cell free culture supernatants were collected at the end of a 4hr co-culture and analyzed for IFNγ release by ELISA (C). The contribution of FcR mediated effector functions of NK cells (i.e. ADCC) on NK cell mediated A431 tumor cell lysis was tested by blocking the FcR receptor on NK cells (D). Data presented is from six individual PBNK donors. Columns are mean of triplicate values; with bars showing SD. Mean ± SD for each significant condition are represented as p = <0.05 *, <0.01 **, <0.005 ***, <0.001 ****.

To establish which of the anti-EGFR mAbs, cetuximab or panitumumab, exerted higher functionality with respect to EGFR recognition and cytotoxicity, both were tested on strongly EGFR positive (EGFR^+++^) A431 cells. Flowcytometric detection of EGFR using biotinylated cetuximab (**Δ**MFI = 217) was nearly two fold intense than observed with biotinylated panitumumab (**Δ**MFI = 123), as shown in Fig 1A and 1B. Next, A431 cells were treated with cetuximab or panitumumab to calculate the concentration required to induce 50% of maximal cytotoxicity. At higher concentrations than 1000μg/ml both mAbs were equally cytotoxic (61 ± 2%). Titrating down, the concentrations required to induce 50% of maximal cytotoxicity (EC_50_ value) of EGFR^+++^targets were found to be 5μg/ml for cetuximab (31 ± 2%) and 100μg/ml for panitumumab (36 ± 1%) respectively (Fig 1C). Based on these findings concentrations of 5μg/ml of cetuximab and 100μg/ml of panitumumab were used in all subsequent experiments to assess their ADCC efficacy when combined with NK cells. To this end A431 cells were coated with either cetuximab or panitumumab and co-incubated with activated NK cells. A significant increase in A431 cell death was observed over NK cells only, when NK cells were combined with cetuximab, but not with panitumumab coated targets (Fig 2A). This is consistent with the IgG_2_ isotype of panitumumab, which precludes high-affinity binding to NK CD16a. In line with these ADCC data, degranulation of NK cells, as assessed by CD107a expression, was significantly increased when tumor target cells were coated with cetuximab (Fig 2B and S2B Fig). Similarly to the observed ADCC and CD107a levels, IFNγ production was increased in NK cell and cetuximab co-cultures (Fig 2C). To formally demonstrate that the cetuximab-related increase in target cell killing was due to ADCC, NK-FcR receptors were blocked using a FcR blocking reagent (Miltenyi Biotec) and then incubated with cetuximab coated target cells. As shown in Fig 2D and S2C Fig, FcR blocking resulted in a considerable and statistically significant reduction in degranulation of the CD16a^+^ NK cell compartment. Together, these data provide a clear rationale to combine NK cells and cetuximab to increase the killing of EGFR^+++^ targets by ADCC.

### NK CD16a (FcγRIIIa) polymorphism does not significantly influence cetuximab induced ADCC *ex vivo*

**Fig 3:**
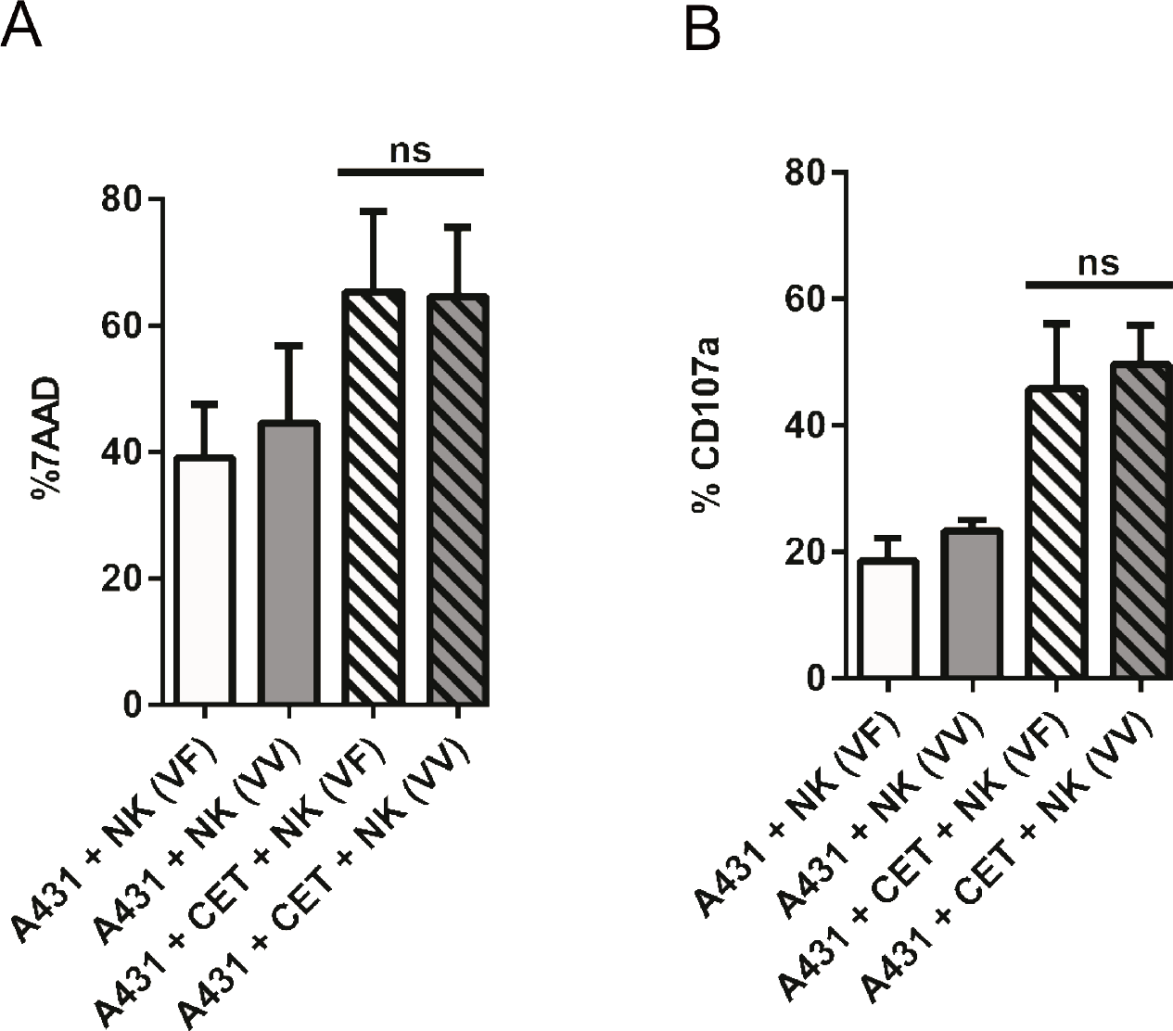
Evaluation of NK cell CD16a polymorphism on cetuximab induced ADCC. Differences in cetuximab induced ADCC between NK cells expressing V/V and V/F polymorphic versions of CD16 were analyzed. Natural cytotoxicity and NK cell CD16a mediated degranulation in the absence and presence of cetuximab coated A431 cells are shown in Fig A and B. Columns are mean of triplicate values; with bars showing SD. Data are from independent experiments performed in triplicates from 3 V/V and 3 V/F donors. Mean ± SD for each significant condition are represented as p = <0.05 *, <0.01 **, <0.005 ***, <0.001 ****.

Several clinical studies reported that NK FcγRIIIa receptor polymorphisms affected the clinical efficacy of cetuximab, due to a variable binding affinity to NK cells, thereby directly affecting the potency of ADCC [15, 16]. In order to test if V158V (V/V) and V158F (V/F)polymorphic versions of CD16 translated to differences in ADCC upon engagement with cetuximab, cytotoxicity assays were performed with NK cells from 6 donors, 3 with the V/V polymorphism and 3 with the V/F polymorphism. Cytotoxicity against A431 and degranulation of the CD16^+^ NK cell fraction was assessed after a 4hr co-culture of cetuximab coated A431 cells and NK cells. No significant differences in target cell death (Fig 3A) or degranulation (Fig 3B) were observed between NK cell donors with V/V and V/F CD16/FcγRIIIa polymorphism.

### NK cells efficiently lyse EGFR^+^/^-^ and RAS^wt^/^mut^ colon cancer cell lines

**Fig 4:**
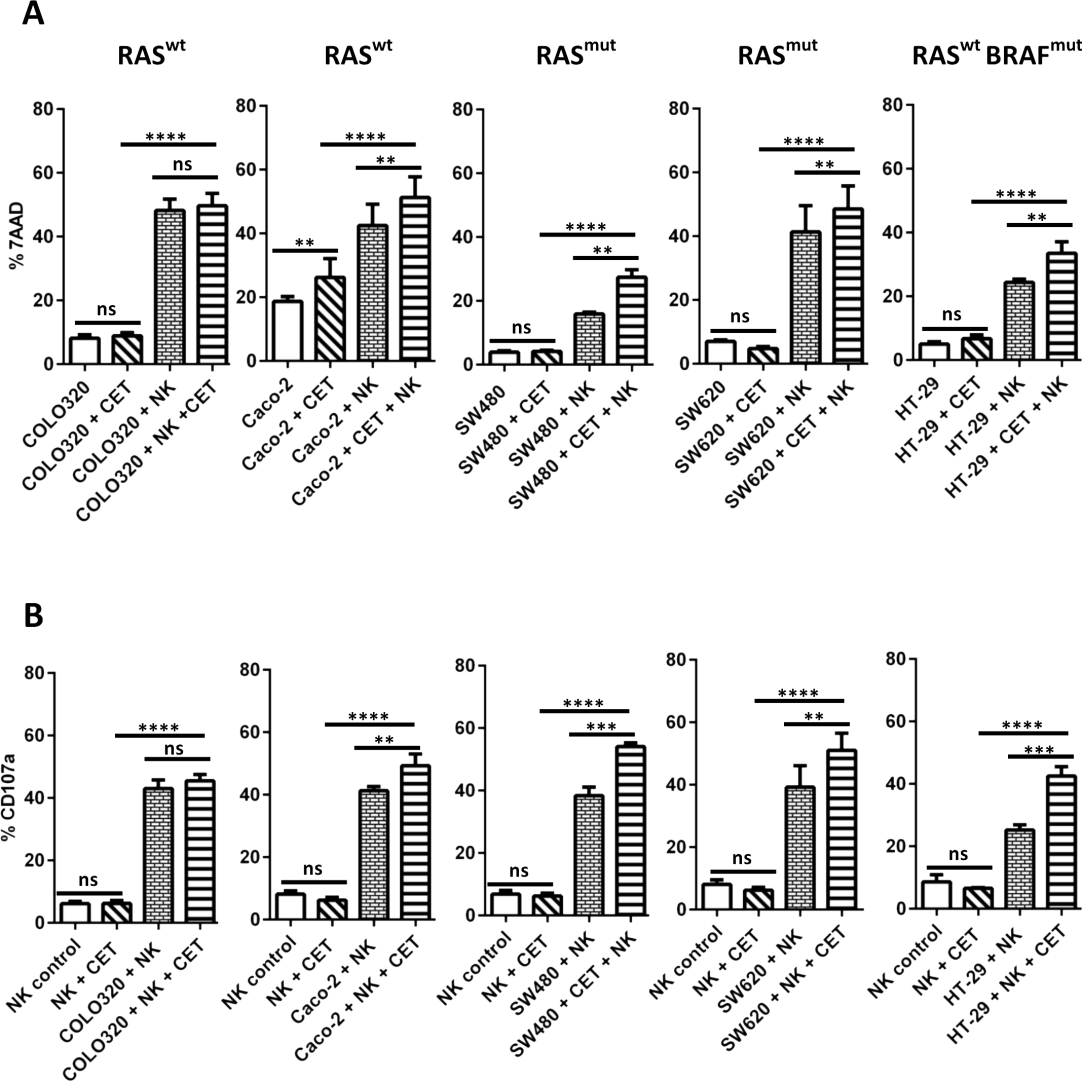
Combinatorial effect of NK cells and cetuximab on EGFR^+/-^, RAS^wt / mut^ and BRAF^mut^ cells. Cytotoxicity effected by cetuximab and NK cells either alone or in combination against COLO320 (EGFR^-^, RAS^wt^), Caco-2 (EGFR^+^, RAS^wt^), SW480 (EGFR^+^, RAS^mut)^, SW620 (EGFR^+^, RAS^mut)^ and HT-29 (EGFR^+^, RAS^wt,^ BRAF^mut)^, was compared between these cell lines measuring target cell death (A). Functional differences between NK cells that were co-cultured with tumor cell lines in the presence or absence of cetuximab were evaluated by studying NK cell degranulation (B). Data presented is from five individual PBNK donors. Columns are mean of triplicate values from five experiments; bars represent SD. Mean ± SD for each significant condition are represented as p = <0.05 *, <0.01 **, <0.005 ***, <0.001 ****.

Having established the anti-tumor efficacy of the cetuximab/NK cell combination with the EGFR^+++^ cell line A431, our next aim was to extend these findings to colon cancer cell lines COLO320 (EGFR^-^, RAS^wt^), SW480 (EGFR^+^ RAS^mut^; KRAS exon 2 c.35>T; p.G12V), SW620 (EGFR^+^ RAS^mut^; KRAS exon 2 c.35>T; p.G12V) and HT-29 (EGFR^+^, RAS^wt^, BRAF^mut^). Binding ability towards biotinylated cetuximab was negative for COLO320 EGFR (ΔMFI = 1), with a relatively low EGFR expression detected on SW480 cells (ΔMFI = 17) as shown in S1A and S1B Fig. The EGFR expression level (ΔMFI) on the other colon cancer cell lines was 23 for CaCo-2, 7 for HT-29, and 3 for SW620 (data not shown). In addition, cetuximab as a single agent could not induce cytotoxicity, even at increasing concentrations of up to 1000μg/ml (S1C and S1D Fig). In the next step, to test NK killing effects alone and in combination with cetuximab, all five colon cancer cell lines were coated with 5μg/ml cetuximab to see if sensitizing target cells with cetuximab could contribute to improved NK cell killing through ADCC. The results showed that COLO320, Caco-2 and SW620 cells are more sensitive for NK killing compared to SW480 and HT-29 cells (Fig 4A). However, while co-incubation with cetuximab did not result in a significant increase in COLO320 tumor cell death, a significant increase in killing was seen in four EGFR^+^ cell lines independent of RAS and BRAF status. With respect to Caco-2, SW480, SW620 and HT-29, the cytotoxic effect could be increased by cetuximab (Fig 4A), this enhancement of the cytolytic NK cell response was also evident from an increased rate of degranulation in the condition where NK cells and cetuximab were added in combination to EGFR^+^ colon cancer cell lines (Fig 4B). Interestingly, we also assessed tumor cell expression of HLA-E and NK cell expression of NKG2A as HLA-E is a known ligand for the NK cell inhibitory receptor NKG2A. As can be seen in the S3A and S3B Fig, while NKG2A levels were comparable on NK cells in all donors that were tested, HLA-E expression levels were strikingly different and correlated with the overall susceptibility of tumor cells to NK cell lysis as shown in Fig 4, where cell lines with low HLA-E levels (COLO320, Caco-2, SW620) were more sensitive to NK cell killing compared to higher HLA-E expressing cell lines (SW480 and HT-29).These data suggest that NK cells have the potential to improve anti-EGFR mAb therapy efficacy even in situations where tumors carry RAS^mut^, BRAF^mut^ or are EGFR^-^.

### NK cells efficiently target and kill primary colon tumor cells

**Fig 5:**
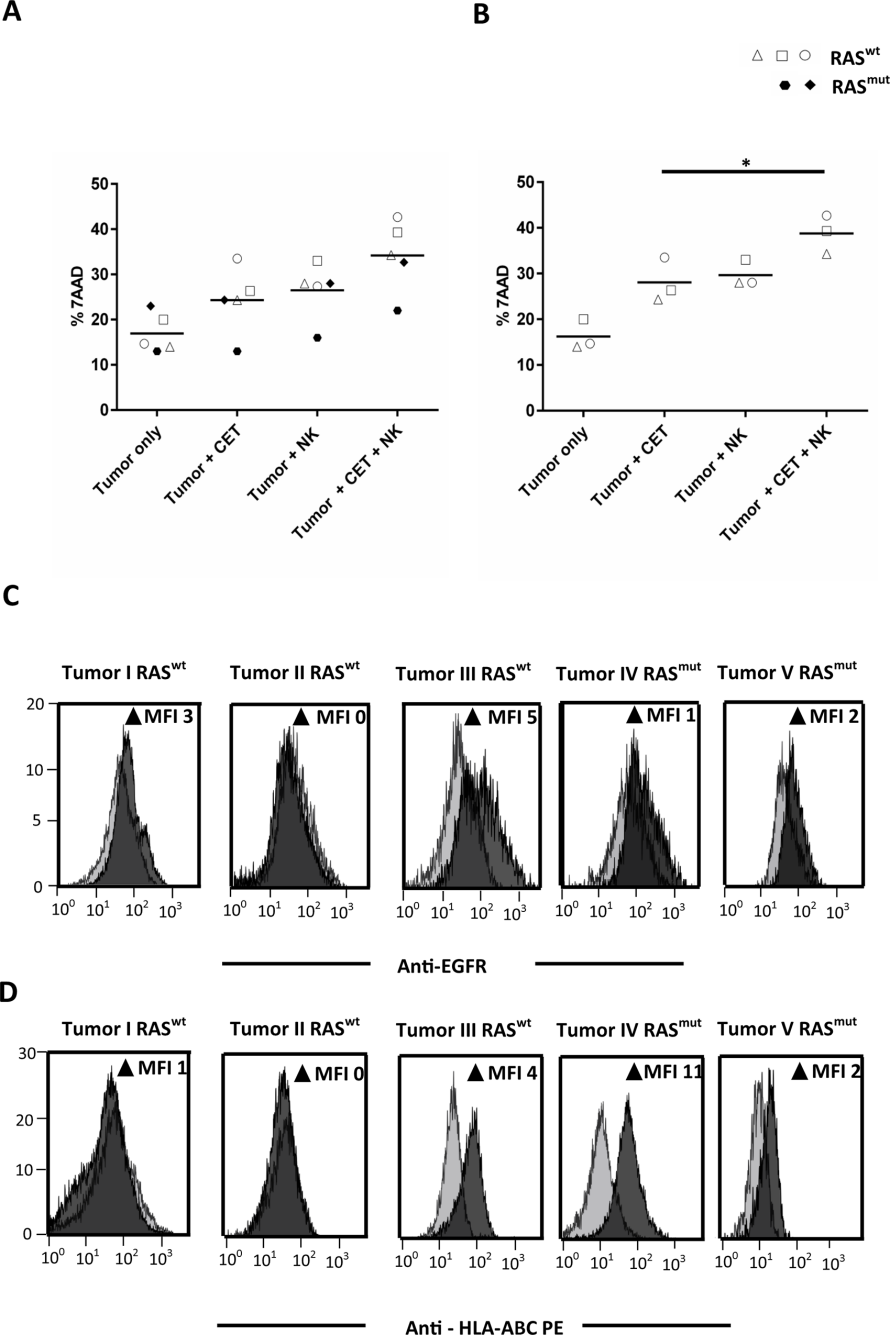
Cetuximab enhances NK cell killing of primary colon tumors through ADCC. Primary colon tumor cells were pretreated with cetuximab and incubated with activated NK cells to monitor ADCC effects. NK cells and cetuximab alone controls were included to document the effects of monotherapy. Percentages of tumor cell death from five CRC patients are shown in Fig A and each symbol represents one specific patient. RAS^wt^ tumors I, II and III are indicated by open squares, triangle and circles, and RAS^mut^ tumors IV and V are shown as closed rhombus and hexagon. Fig B shows only the cytotoxicity data of RAS^wt^ tumors. All five primary tumors used in the cytotoxicity assay were tested for EGFR and HLA-ABC expression as shown in Fig C and D. Grey histograms represent the isotype controls, black shades represent EGFR and HLA-ABC expression for tumor samples I-V. Data points from Fig A and B are mean of triplicate values from five patients; each significant condition are represented as p = <0.05 *, <0.01 **, <0.005 ***, <0.001 ****.

As an indication that our observations regarding combined cetuximab/NK cell antitumor efficacy could be extrapolated to the clinical situation, primary tumor material from five patients with CRC was subjected to NK cell killing in the presence or absence of cetuximab in an experimental set-up that was essentially the same as used for the cytotoxicity experiments with colon cancer cell lines described above. In all 5 patients, tumor cells were effectively lysed by NK cells, but only 3 out of 5 tumors responded to cetuximab monotherapy. Importantly, and as shown in Fig 5A and 5B, cetuximab increased NK cell tumor cell lysis regardless of whether tumors were susceptible to cetuximab monotherapy. The differences in response to cetuximab monotherapy were related to the RAS mutation status of the tumors as the 2 tumors not responding to cetuximab monotherapy had a mutation (KRAS exon 2, c.35>T; p.G12V) in the RAS gene. Fig 5C and 5D show tumor cell EGFR and HLA-ABC expression levels respectively; all tumors tested had low levels of EGFR and HLA-ABC. Together, these experiments have demonstrated that NK cells have the ability to kill primary tumor cells, and that this can be further increased via cetuximab-mediated ADCC in both RAS^wt^ and RAS^mut^ tumors.

## Discussion

Anti-EGFR therapies currently in practice are not adequate to prevent mCRC. The main aim of this study was to determine whether NK cells and cetuximab could be combined to improve their anti-tumor efficacy and widen their applicability in mCRC independent of EGFR and RAS status. NK cells are the immune effectors of choice for ADCC induced by cetuximab [17, 18]. Higher frequencies of NK cells in the peripheral blood and increased NK tumor infiltration have both been associated with a favorable prognosis in CRC [19]. The ability of NK cells to target cancer stem cells has also been reported [20]. Thus, the presence of an adequate number of cytolytic NK cells has the potential to drive potent ADCC in combination with cetuximab.

EGFR is overexpressed in various solid tumors, making it an attractive biomarker for anti-EGFR therapy [21]. Cetuximab, when administered following chemotherapy or in chemo-refractory RAS^wt^ mCRC patients improves their progression-free survival [22]. However, although the ensuing anti-tumor response is reliant on the presence of a non-mutated RAS gene, the overall response rate of cetuximab is still only around 23%, even among patients with RAS^wt^ tumors [23–25]. We hypothesized that NK cells in combination with cetuximab could enhance the cytotoxic effects of cetuximab in EGFR^+^ RAS^wt/mut^ BRAF^mut^ CRCs, besides NK cell natural cytotoxicity can mediate anti-tumor activity on EGFR^-^ CRCs that do not respond to anti-EGFR therapy.

In our experiments, a group of solid tumor cell lines was selected based on EGFR expression, RAS and BRAF status. Titrating cetuximab in cultures of A431 cells (EGFR^+++^, RAS^wt^), SW480 cells (EGFR^+^, RAS^mut^) and COLO320 cells (EGFR^-^, RAS^wt^) demonstrated that cetuximab alone induced cytotoxicity only in EGFR^+^ RAS^wt^ tumor cells (Fig 2A and S1C and S1D Fig). In a next set of experiments, NK cells were combined with cetuximab and this was shown to enhance the lysis of EGFR^+++^ RAS^wt^ tumor cells. Using NK-FcR blocking assays this enhancement was shown to be mediated through ADCC (Fig 2D and S2A–S2C Fig). Investigating the role of NK cell CD16a (FcγRIIIa) polymorphisms in relation to cetuximab efficacy, we did not see a statistically significant difference between V/F and V/V donors with respect to the induction of NK cell degranulation or tumor target cell lysis. These data are in line with the recent clinical study observations published by Mellor et al, concluding that FcγRIIIa genotype differences were not predicting significant therapeutic benefits for cetuximab [26].

Of interest, our data demonstrated that NK cells were capable of also killing EGFR^-^ RAS^wt^ cells, EGFR^+^ RAS^wt^, EGFR^+^ RAS^mut^ and EGFR^+^ BRAF^mut^ cells. Furthermore, though cetuximab was completely ineffective as monotherapy against SW480 (EGFR^+^ RAS^mut^), SW620 (EGFR^+^ RAS^mut^) and HT-29 (EGFR^+^ RAS^wt^ BRAF^mut^) cells, cetuximab could increase the target cell killing of NK cells in this setting. The ability of NK cells to lyse tumor targets independent of EGFR, RAS and BRAF status combined with the observation that cetuximab can enhance this cytotoxic effect in EGFR expressing tumors regardless of RAS and BRAF mutational status is an added advantage as it can result in effective target cell lysis of tumors responsive or non-responsive to cetuximab monotherapy. In the clinics, the eligibility criteria for anti-EGFR therapy is based on RAS^wt^ status, whereas the CRC patients are not evaluated for EGFR expression levels; the fact that NK cells can effectively target EGFR^-^ tumor cells could provide an ideal platform to treat metastatic CRC patients having variable levels of EGFR expression.

We observed that although NK cells can efficiently kill EGFR^+/-^ RAS^wt / mut^ cells (A431, COLO320, Caco-2 and SW620), its efficacy was relatively low on EGFR^+^ RAS^mut^ SW480 and EGFR^+^ RAS^wt^ BRAF^mut^ HT-29 cells. This difference could be related to the differential expression of the inhibitory non-classical HLA-E; i.e. RAS^mut^ SW480 and BRAF^mut^ HT-29 cells were found to have high expression of HLA-E, an inhibitory ligand for the NK cell inhibitory receptor NKG2A (S3 Fig), making them less susceptible to NK cell killing [27–29]. Of interest, though NK cell induced tumor cell lysis was still lowest in SW480 and HT-29, cetuximab appeared to at least in part bypass the inhibitory effect mediated through NKG2A/HLA-E interactions, probably by tipping the balance of activating and inhibitory signals more towards NK cell activation through binding of cetuximab to the activating FcγRIIIa. Furthermore, blocking HLA-E on SW480 and HT-29 cells could pave the way for more effective NK cell killing and could hence translate into superior cell death when combined with cetuximab in this setting [30, 31].

From the data obtained using primary colon cancer cells as target cells, it was evident that NK cells could kill primary tumor cells and that while cetuximab monotherapy was effective only in RAS^wt^ tumor samples, NK cell cytotoxicity could be increased by cetuximab irrespective of RAS mutational status, making these primary tumor cells ideal targets for observing the potentiating effect of cetuximab induced ADCC on NK cell natural cytotoxicity against EGFR^low^ primary tumors (Fig 5A and 5B) [32]. Menon et al, showed that HLA Class-I was down-regulated in 72% of patients with CRC [33], thereby making the majority of CRC cells highly susceptible to NK cell mediated killing. The clinical relevance of FcR polymorphisms and NK ADCC influencing the treatment of RAS mutated mCRC patients have been studied by Bibeau and his team reporting disease stabilization in 10 out of 27 patients with RAS mutant tumors following cetuximab monotherapy [34]. In accordance with their findings, our in vitro data support the notion that NK cell ADCC could exert relevant cytotoxicity in this setting. Of note, clinical studies point out that most of the cancer patients present with an imbalance in their immune subsets, which tilts the balance towards tumor progression. Additionally, adoptive transfer of ex vivo activated autologous NK cells has failed to demonstrate clinical responses in mCRC patients [35–37]. Combining all these factors, allogeneic NK cell transplantation might be the preferred choice to treat solid tumors and hence support the immune system of the patient with sufficient cytolytic NK cells to mount a strong anti-tumor attack. Furthermore, adoptive transfer of large number of allogeneic cytolytic NK cells could be an option to strengthen tumor eradication in combination with cetuximab on RAS mutated tumors via ADCC. Allogeneic NK cell therapies are widely explored over recent years due to their cytotoxic nature providing a window to induce a HLA-mismatch setting to efficiently target tumor cells [38]. Different sources of allogeneic NK cell products are currently in use for clinical applications from adult PBNK cells [39], umbilical cord blood stem cell derived NK cells [40, 41] and engineered NK cell lines [42]. Adoptive transfer of large number of allogeneic NK cells could repopulate the immune system, providing sufficient numbers of cytolytic NK cells to support ADCC with cetuximab. An increased number of NK cells in the blood stream could also target circulating tumor cells preventing metastasis and with a further role in decreasing or removing residual tumor load [43]. Further clinical confirmation of NK cell adoptive transfer advantages in combination with cetuximab on RAS^mut^ or BRAF^mut^ mCRC tumors is warranted and calls for clinical trials.

In conclusion, the ability of functional NK cells to overcome the limitations of anti-EGFR therapy has been clearly demonstrated. The availability of allogeneic NK cells, combined with cetuximab could pave the way to demonstrate the therapeutic efficacy of this approach in patients with RAS^mut^, BRAF^mut^ and EGFR^low/-^ CRC.

## Materials and Methods

### Cell lines

Cell lines A431, COLO320, SW480, Caco-2, SW620, HT-29 were obtained from ATCC and cultured in Dulbecco’s modified medium (DMEM; Invitrogen, Carlsbad CA, USA) containing 100 U/ml penicillin, 100 μg/ml streptomycin and 10% fetal calf serum (FCS; Integro, Zaandam, The Netherlands) Cell cultures were passaged every 5 days. Cultures were maintained in a 37°C, 95% humidity, 5% CO_2_ incubator.

### Peripheral blood NK cell isolation and activation from whole blood specimens

Mononuclear cells (MNCs) were isolated from peripheral blood using Lymphoprep™ (STEMCELL Technologies, The Netherlands) density gradient centrifugation from buffy coats obtained from anonymous healthy blood donors (Sanquin Blood Supply, Amsterdam) with written informed consent for research use, in accordance with the ‘‘Code for Proper Use of Human Tissues” as formulated by the Dutch Federation of Medical Scientific Organizations (www.fmwv.nl) [44]. CD56^+^ NK cells were isolated from MNCs using a MACS Human NK cell isolation kit (Miltenyi Biotech, Bergisch Gladbach, Germany) according to the manufacturer’s instructions. The cell number and purity of the isolated NK cell fraction were analyzed by flow cytometry. Isolated NK cells were activated overnight with 1000U/ml IL-2 (Proleukin^®^; Chiron, München, Germany) and 10ng/ml IL-15 (CellGenix) for use in cytotoxicity assays. NK cell purity and viability were checked using CD3 PE, 7AAD (BD Biosciences), CD56 APC Vio 770, and CD16 APC (Miltenyi Biotech). The parameters compared before and after activation were NK purity (CD56^+^%, 83 ± 9% vs. 82 ± 9%), NK CD16% 88 ± 10% vs 85 ± 11%) and NK viability (91 ± 3% vs 86 ± 2%) respectively.

### Anti- EGFR monoclonal antibodies used for the cytotoxicity assay

The anti-EGFR mAbs cetuximab (Erbitux®) and panitumumab (Vectibix®) were purchased through the VU University medical center pharmacy.

### Biotinylation of anti-EGFR mAbs

To assess the binding of cetuximab and panitumumab to EGFR expressing target cells, anti-EGFR mAbs were concentrated using Amicon ultra centrifuge 0.5ml 30K tubes (EMD Millipore, Netherlands). Concentrations of the mAbs were adjusted to 20mg/ml and then biotinylated using Biotin-N-hydroxysuccinimide ester (Sigma Aldrich, St Louis, USA) according to the manufacturer’s instructions. The biotinylated anti-EGFR mAbs were incubated with A431 (1x10^6^) cells for 1hr, washed twice in ice cold PBS and stained with streptavidin APC (BD biosciences, Netherlands). A nonspecific IgG_1_ and IgG_2_ specific APC labeled antibody was used as a negative control.

### DNA extraction and FCGR3A V158V and V158F (V/V and V/F) polymorphism genotyping

Genomic DNA (gDNA) was isolated from MNCs using QIA amp DNA kit (QIAGEN, Westburg, The Netherlands). Purified DNA was eluted in a volume of 100μl. Purity and Quantity of gDNA was measured using the nanodrop method (NANODROP 1000, Thermo Scientific). About 40–200ng of gDNA was used for FcγRIIIa polymorphism assays. FcγRIIIa primers ID: C__42463377_10 were purchased from Life Technologies, The Netherlands. 40ng of gDNA, 6.25μl of PCR master mix and 12.5μl mix of forward and reverse primers with VIC and FAM labeled probes for V/V and V/F polymorphism respectively were added. Readings were interpreted using v2.0.1 software (Bio Rad, Netherlands). Known controls for VV and VF genotypes were included in the experiment.

### Flow cytometry

Flow cytometry analysis was done on a BD LSRFORTESSA X-20 (BD Biosciences). Cell numbers and expression of cell-surface markers were determined by flow cytometry. The cell numbers and the population of live cells was determined by gating on CD45^+^ cells based on forward scatter (FSC) and side scatter (SSC). For analysis of phenotype, the cells were gated only on FSC/SSC and further analyzed for the specific antigen of interest. Cells were incubated with the appropriate concentration of antibodies for 30 min at 4°C. After washing, cells were suspended in FACS buffer.

### Flow cytometry-based cytotoxicity and degranulation studies

Flow cytometry was used for the read-out of cytotoxicity assays. Target cells were labeled with 5μM pacific blue succimidyl ester (PBSE; Molecular Probes Europe, Leiden, The Netherlands) in a concentration of 1x10^7^ cells per ml for 10 min at 37°C. The reaction was terminated by adding an equal volume of FCS, followed by incubation at room temperature for 2 min after which stained cells were washed twice with 5 ml DMEM/10% FCS. After washing, cells were suspended in DMEM/10% FCS to a final concentration of 5 x 10^5^/ml. CD56^+^ NK cells were washed with PBS and suspended in Glycostem Basal Growth Medium (GBGM) + 2% FCS to a final concentration of 5 x 10^5^/ml. Target cells were co-cultured with effector cells at an E:T ratio of 1:1 in a total volume of 250 μl in 96-wells flat-bottom plates (5 x 10^4^ targets in 100 μl of DMEM + 10% FCS incubated with 5 x 10^4^ effectors in 100 μl of GBGM + 2% FCS, further supplemented with 25 μl of GBGM + 2% FCS and DMEM + 10% FCS medium). NK cells and target cells alone were plated out in triplicate as controls. Target cells were coated with anti-EGFR mAbs for 1h at 4°C. Cells (A431, COLO320, Caco-2, SW80, SW620 and HT-29) were washed and co cultured with activated NK cells. To measure degranulation by NK cells, anti-CD107a PE (Miltenyi Biotech, Germany) was added in 1:20 dilution to the wells. After incubation for 4h at 37°C, 75 μl supernatant was collected and stored at -20°C for analysis of cytokine production. Cells in the remaining volume were harvested and stained with 7AAD (1:20). Degranulation of NK cells was measured by detecting cell surface expression of CD107a. After 4 hrs of incubation at 37°C, CD56 APC Vio 770 (1:25) and CD16 APC (1:25) (Miltenyi Biotech, Germany) were added to the co-cultures and NK CD107a degranulation was measured for CD56^+^ NK, CD56^+^CD16^+^ NK and CD56^+^CD16^-^ NK cells.

### IFNγ production assay

Production of IFNγ by target cell-stimulated NK cells was measured in the supernatant of the co-cultures by ELISA (Sanquin, Amsterdam, The Netherlands). Absorbance was measured at 450 nm with a Multiscan MCC/340 ELISA reader (Titertek, Huntsville, Alabama, USA).

### Primary colon tissue dissociation and storage

Colon cancer tissues from patients participating in trials conducted at the VU University medical center in Amsterdam, The Netherlands were collected and processed as described [45] after written informed consent and used for this study according to protocols approved by the VUmc IRB (IRB00002991; IORG number 0002436) [46]. Tumor material was further confirmed histologically and were screened for HLA class I (Clone W6/32), and EGFR expression. Tumor tissues were washed thrice, scraped, cut into small fragments and digested mechanically using collagenase A (Roche Diagnostics, The Netherlands) based growth medium. Digestion was carried out in a sterile glass flask with continuous stirring for 45 mins. This step was repeated twice and the single cell suspension was collected through a 45μm sterile filter. Cells were counted and then frozen under controlled conditions in liquid nitrogen. Primary tumor cells were thawed, ficoll based density grade separation was done to remove dead cells and finally resuspended in DMEM +10% FCS medium for the cytotoxicity assays.

### RAS typing

The mutational status of *KRAS* exon 2/3/4, *NRAS* exon 2/3/4 and *BRAF* exon 15 was assessed by high resolution melting (HRM) assay followed by Sanger sequencing of HRM-PCR products with an aberrant melt curve, essentially as described previously [47, 48].

### Statistical analysis

Statistical analysis was performed using Graph Pad Prism software. Differences between conditions were determined using two way anova with multiple comparisons between column means. Results from cytotoxicity experiments are described as mean ± standard deviation of the mean (SD). A p-value of <0.05 was considered statistically significant.

## Supporting Information

S1 Fig Cetuximab cytotoxic activity against EGFR^+^ RAS^mut^ and EGFR^-^ RAS^wt^ colon cancer cells. SW480 and COLO320 cells were incubated with 100μg/ml of biotinylated cetuximab for 1hr at 4°C and analyzed for their EGFR recognition by cetuximab. A and B, Histograms showing binding of cetuximab to (A) COLO320 and (B) SW480 cells. Grey shades represent the streptavidin APC control, black shades represent binding of cetuximab. Further, SW480 and COLO320 cells were stained with PBSE and exposed to increasing concentrations of cetuximab for 1hr at 4°C after which unbound antibodies were removed and cells were cultured for an additional 4hrs at 37°C. Target cell death was determined by assessing the percentage of 7AAD positive COLO320 (C) and SW480 (D) cells at the end of incubation.
(TIF)

S2 Fig Degranulation of NK cell CD56^bright^ and CD56^dim^ subsets in response to cetuximab coated tumor target cells. A representative example of PBNK degranulation pattern upon target exposure to A431 tumor targets in the presence or absence of cetuximab. CD56^bright^ and CD56^dim^ degranulate upon target cell recognition (Fig A), with an increase in degranulation in CD56^dim^ CD16^+^ subset of NK cells when target cells are coated with cetuximab (Fig B). The degranulation can be reduced by blocking Fc receptors (Fig C) and this also decreases the degranulation in the CD56^bright^ subset of NK cells.
(TIF)

S3 Fig Expression of HLA-E on tumor cell lines and NKG2A on NK cells used for cytotoxicity experiments. Surface expression of HLA-E on COLO320, Caco-2, SW620, SW480 and HT-29 and NK cell NKG2A expression levels of five healthy donors used for the cytotoxicity assays were determined by flow cytometry as shown in Fig A and B. Columns are mean of triplicate values from two independent experiments, bars represent SD. Mean ± SD for each significant condition are represented as p = <0.05 *, <0.01 **, <0.005 ***, <0.001 ****.
(TIF)
