# Supplementary figures and images for: Combination of NK Cells and Cetuximab to Enhance Anti-Tumor Responses in RAS Mutant Metastatic Colorectal Cancer
Source: PLoS One. 2016 Jun 17;11(6):e0157830. doi: 10.1371/journal.pone.0157830 (PMC4912059; doi:10.1371/journal.pone.0157830)

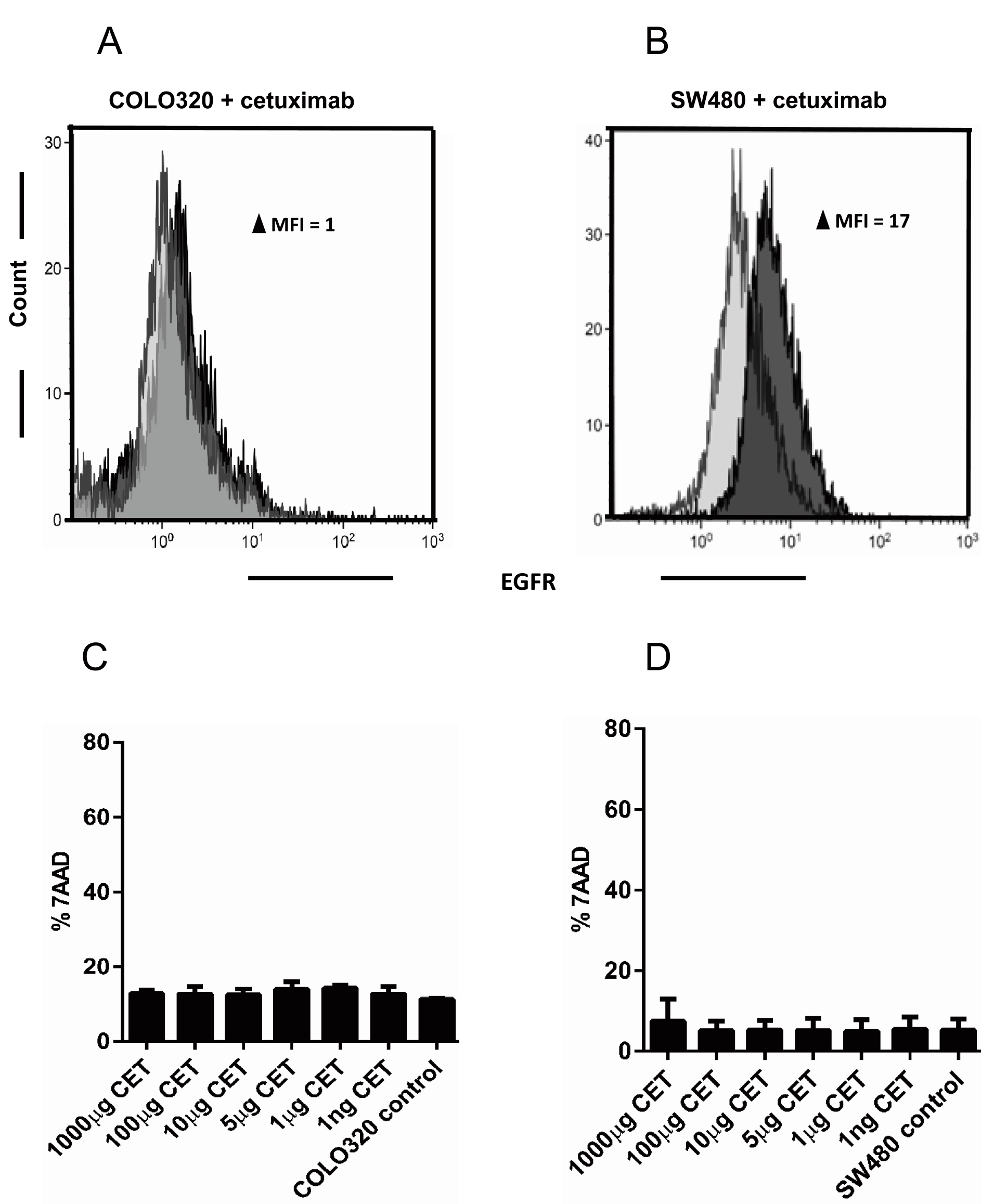

Supplement: S1 Fig — SW480 and COLO320 cells were incubated with 100μg/ml of biotinylated cetuximab for 1hr at 4°C and analyzed for their EGFR recognition by cetuximab. A and B, Histograms showing binding of cetuximab to (A) COLO320 and (B) SW480 cells. Grey shades represent the streptavidin APC control, black shades represent binding of cetuximab. Further, SW480 and COLO320 cells were stained with PBSE and exposed to increasing concentrations of cetuximab for 1hr at 4°C after which unbound antibodies were removed and cells were cultured for an additional 4hrs at 37°C. Target cell death was determined by assessing the percentage of 7AAD positive COLO320 (C) and SW480 (D) cells at the end of incubation. (TIF) [file pone.0157830.s001.tif]

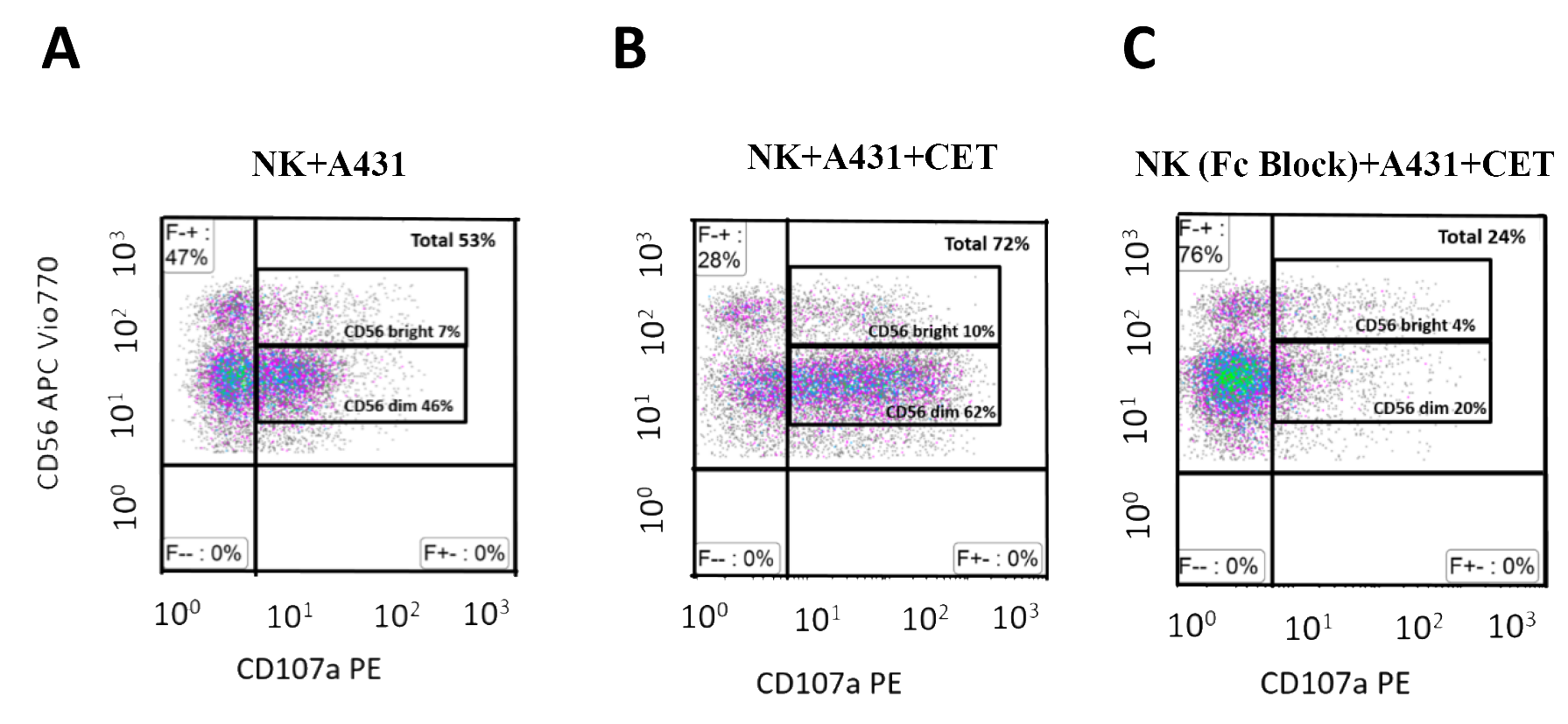

Supplement: S2 Fig — A representative example of PBNK degranulation pattern upon target exposure to A431 tumor targets in the presence or absence of cetuximab. CD56bright and CD56dim degranulate upon target cell recognition (Fig A), with an increase in degranulation in CD56dim CD16+ subset of NK cells when target cells are coated with cetuximab (Fig B). The degranulation can be reduced by blocking Fc receptors (Fig C) and this also decreases the degranulation in the CD56bright subset of NK cells. (TIF) [file pone.0157830.s002.tif]

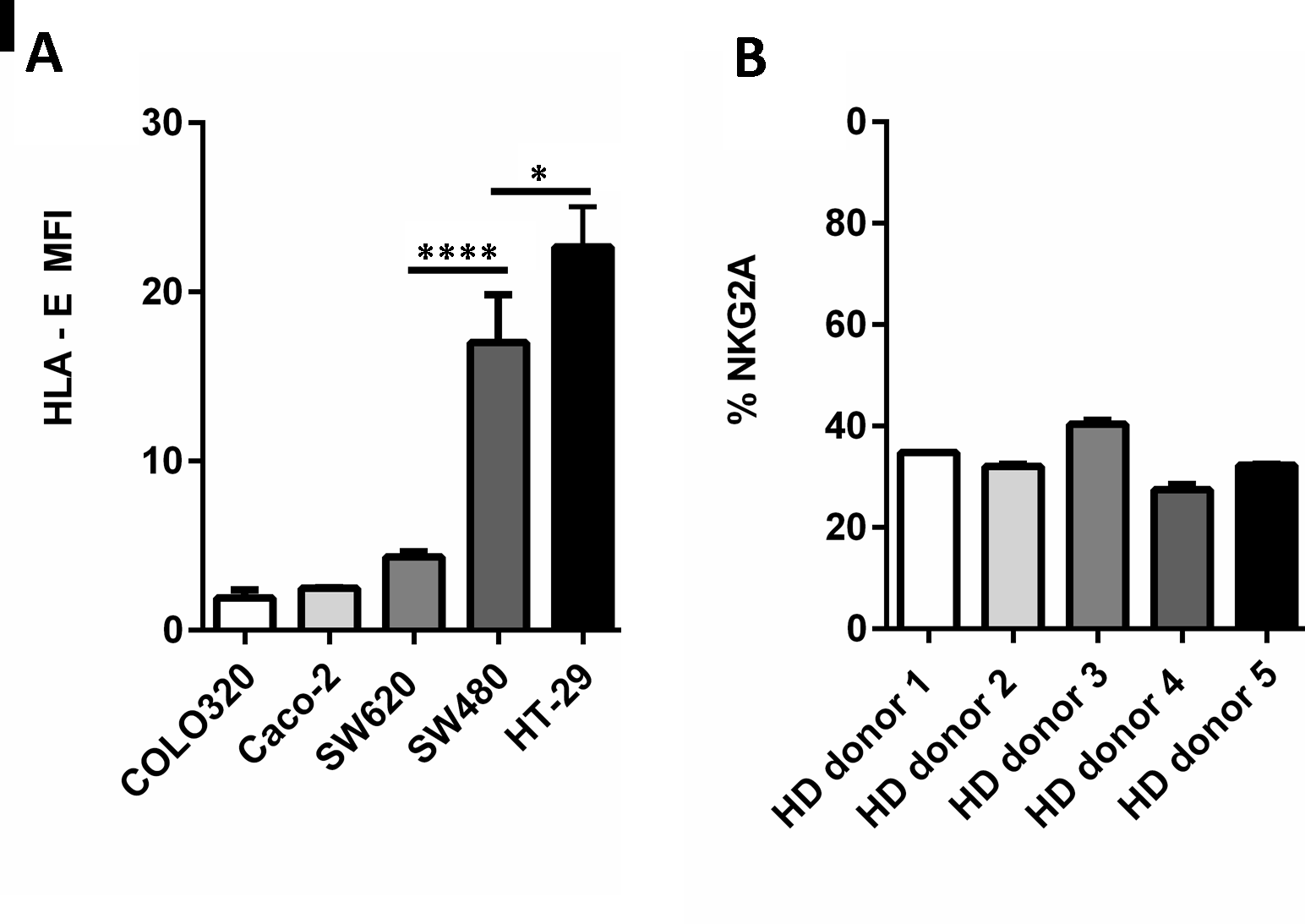

Supplement: S3 Fig — Surface expression of HLA-E on COLO320, Caco-2, SW620, SW480 and HT-29 and NK cell NKG2A expression levels of five healthy donors used for the cytotoxicity assays were determined by flow cytometry as shown in Fig A and B. Columns are mean of triplicate values from two independent experiments, bars represent SD. Mean ± SD for each significant condition are represented as p = <0.05 *, <0.01 **, <0.005 ***, <0.001 ****. (TIF) [file pone.0157830.s003.tif]
